# Biomarkers for Early Diagnosis and Prognosis of Malignant Pleural Mesothelioma: The Quest Goes on

**DOI:** 10.3390/cancers10060203

**Published:** 2018-06-15

**Authors:** Caterina Ledda, Paola Senia, Venerando Rapisarda

**Affiliations:** Occupational Medicine, Department of Clinical and Experimental Medicine, University of Catania, Catania 95123, Italy; paosenia@hotmail.it (P.S.); vrapisarda@unict.it (V.R.)

**Keywords:** asbestos, exposure, occupational cancer

## Abstract

Malignant pleural mesothelioma (MM) is a highly aggressive tumor characterized by a poor prognosis. Although its carcinogenesis mechanism has not been strictly understood, about 80% of MM can be attributed to occupational and/or environmental exposure to asbestos fibers. The identification of non-invasive molecular markers for an early diagnosis of MM has been the subject of several studies aimed at diagnosing the disease at an early stage. The most studied biomarker is mesothelin, characterized by a good specificity, but it has low sensitivity, especially for non-epithelioid MM. Other protein markers are Fibulin-3 and osteopontin which have not, however, showed a superior diagnostic performance. Recently, interesting results have been reported for the HMGB1 protein in a small but limited series. An increase in channel proteins involved in water transport, aquaporins, have been identified as positive prognostic factors in MM, high levels of expression of aquaporins in tumor cells predict an increase in survival. MicroRNAs and protein panels are among the new indicators of interest. None of the markers available today are sufficiently reliable to be used in the surveillance of subjects exposed to asbestos or in the early detection of MM. Our aim is to give a detailed account of biomarkers available for MM.

## 1. Malignant Pleural Mesothelioma

Malignant pleural mesothelioma (MM) is a neoplastic disease strongly associated with asbestos exposure that occurs in many of the serous membranes, predominantly of the pleura and the peritoneum, and to a lesser degree the pericardium and the tunica vaginalis testis [1,2]. It is a highly aggressive cancer with poor prognosis [2]. In fact, the median survival rate is less than 12 months and most patients die within 10–17 months from the onset [3]. Once rare, the incidence of MM has been growing in industrialized countries as a result of extensive exposure to asbestos fibers and it is expected that the incidence will increase in the coming years, especially in developing countries where asbestos is still used, such as Russia, China and India [4,5,6,7]. Approximately 80% of MM can be attributed to asbestos fiber exposure; the six types of minerals that form the fibers that have been marketed with the term asbestos include the serpentine, mineral crisolite and the cummingtonite amphibole fibers, grunerite, actinolite, antofillite, riebeckite and tremolite [5]. Other potential factors for the onset of MM are: the exposure to simian virus 40 (SV40) [8,9], radiation exposure, especially high-dose radiotherapy treatment for lymphomas or other chest malignancies and exposure to eronite, natural and mineral asbestiform fibers [10,11,12,13,14,15,16]. An emerging problem is exposure to non-commercial asbestos fibers and other types of mineral fibers present in rural development areas and in the desert, which have the same health effects as asbestos. The best known of these are the fibers of the mineral eronite, a natural and mineral fiber found in soil and rock that has been associated with high rates of MM, especially in Anatolia (Turkey) [17].

Similarly, the environmental exposure to the Fluoro-edenite mineral fibers, an amphibolic fiber that is found in the Etna volcanic rock, extracted in local quarries and used in construction, has been associated with an increase in MM cases among the population of Biancavilla in Sicily, Italy [18,19,20,21,22,23].

Antigorite mineral fibers have been associated with a cluster of MM in New Caledonia, found in the gravel used for road paving. When asbestos or other natural fibers of asbestos (NOA) are present in the environment, the whole general population is exposed [24,25,26].

The mechanism of MM carcinogenesis induced by asbestos has not been fully understood. Human mesothelial (HM) cells are very susceptible to asbestos cytotoxicity and several pathogenic events may contribute to carcinogenesis during the long latency period between asbestos exposure and tumor onset. Recent work has shown the critical role of TNF-alpha (Tumor Necrosis Factor) of nuclear transcription factor NF-κB, in the response of HM cells to asbestos [27,28]. Crocidolite causes macrophages to accumulate in the pleura and lungs; these macrophages release the TNF-α when they come into contact with the fibers. Crocidolite also induces HM cells to express the TNF-α receptor, TNF-R1, as well as to secrete TNF-α which in its turn activates NF-κB that increase HM cell survival. TNF-α activation of the NF-κB pathway allows mesothelial cells, with DNA damage induced by asbestos, to duplicate rather than die, and if there is sufficient specific genetic damage, HM cells can develop into MM [29,30,31,32,33].

As for the pathological-anatomical classification, MM is classified into three histological subtypes: epithelioid, sarcomatoid, biphasic. The epithelioid subtype characterizes about 70% of all MMs and is less aggressive than the sarcomatoid type, which is highly resistant to chemotherapy and associated with poor survival [34,35,36]. The biphasic subtype has intermediate characteristics and probably corresponds to a transition between the other two histological subtypes. However, the differential MM diagnosis is challenging, because the MM morphology is similar to other tumors. In the MM epithelioid subtype, morphology can be confused with that of non-small cell lung carcinomas, renal cell carcinomas and others. The morphology of the biphasic MM may be similar to that of the synovial sarcomas and other biphasic ones while the sarcomatoid MM is often morphologically indistinguishable from other spindle cell tumors, including carcinosarcoma [34,35,36]. Uncertain diagnostics is a serious and critical problem because patients with various cancers require different treatments and may have a different prognosis. The accuracy of MM diagnosis has been improved using a series of immune-histochemical markers (IHC), including mesothelial markers (calretinin, the most sensitive and WT-1, the most specific) and carcinoma-related markers (CEA, CD15, Ber-EP4, MOC-31, TTF-1) for differential diagnosis. Combining the results obtained with these markers together, it is possible to obtain a more reliable diagnosis [36,37,38,39,40]. In the light of secondary prevention, our attention has been focused on the research of non-invasive biological indicators that allow an early diagnosis of MM and that can be applied to subgroups of high-risk populations, such as former subjects exposed to asbestos.

Biomarkers have been proposed as an effective means for the management of cancer and their search for early detection of MM has been going on for about 30 years [41,42,43]. In an appropriate clinical setting, tumor biomarkers can play a significant role in diagnosis, prognosis, prediction of treatment responses, response monitoring and screening for early illness detection. A good marker should have some important characteristics such as: minimum invasiveness (it should be measurable in easily obtainable biological liquids such as blood), high specificity to avoid false positive results in healthy subjects, sufficient sensitivity to identify subjects affected by MM and ability to discriminate between healthy and sick and between different pathologies when applied in addition to diagnostic imaging.

The promising candidate protein biomarkers, potentially usable in the early diagnosis of MM, are Mesothelin, Osteopontin, Fibulin-3, HMGB1 protein and Aquaporin 1. In the future, there will be molecular markers (i.e., microRNA) in order to enhance MM prevention and diagnosis.

## 2. Osteopontin

Osteopontin, also known as bone sialoprotein I (BSP-1 or BNSP), early T-lymphocyte activation (ETA-1), secreted phosphoprotein 1 (SPP1), 2ar and Rickettsia resistance (Ric), is a protein that in humans is encoded by the *SPP1* gene (secreted phosphoprotein 1), which mediates cell-matrix interaction and cell signaling through interaction with integrin and CD44 receptors [44,45]. Scientific studies have shown that osteopontin is overexpressed in cells exposed to asbestos in vitro, as well as in animal models of carcinogenicity induced by asbestos [46,47]. One of the most important studies of the role of osteopontin in MM was made by Pass et al., who compared 69 patients with benign asbestos-related lung disease to 45 patients without exposure to asbestos and 76 pleural MM surgically treated patients [45]. The results showed that osteopontin serum levels were significantly higher in patients with pleural MM than those with simple asbestos exposure (*p* < 0.001). In particular, with a threshold value of 48.3 ng/mL, the ROC (Receiver Operating Characteristic) curve in the group exposed to asbestos compared to the MM group had a sensitivity of 77.6% and a specificity of 85.5%. A further subgroup analysis showed that at the threshold value of 62.4 ng/mL, the ROC curve comparing patients to stage I MM and patients with asbestos exposure showed a sensitivity of 84.6% and a specificity of 88.4%. Unfortunately, the good results obtained by this study followed numerous others that in some cases confirmed the diagnostic accuracy shown by Pass [45,48,49] but not in others that demonstrate as Osteopontin is a not specific marker for MM [50]. Some potential explanations include the different ELISA (enzyme-linked immunosorbent assay) assays used for osteopontin and the choice of different control populations used, which are not always entirely appropriate for high-risk populations. However, the lack of further validation of the results initially obtained by Pass [45] means that the validity or otherwise of osteopontin as a biomarker of MM is still under discussion.

Hu, Z.D. and colleagues carried out a systematic review and meta-analysis for evaluating circulating levels of osteopontin in the diagnosis of MM [46]. They included six studies in the analysis. The overall diagnostic sensitivity and specificity were 0.65 (95% CI: 0.60–0.70) and 0.81 (95% CI: 0.78–0.85), respectively. The area under summary receiver operating characteristic (sROC) curves (AUC) was 0.83. The diagnostic accuracy of serum and plasma osteopontin was comparable.

Despite the controversies on the diagnostic value of osteopontin, several studies have investigated the prognostic potential and obtained encouraging results. Cappia and colleagues studied the osteopontin immunohistochemical (IHC) expression in short-term survivors and long-term treatment of MM [49]. For this study, 32 long-term survivors (>24 months) and a random sample of 69 short-term survivors (≤24 months) were matched according to the major clinicopathologic characteristic. Osteopontin expression was significantly lower in long-term compared to short-term survivors (*p* < 0.0001), and overall survival analysis showed that low osteopontin expression was associated with longer survival; multivariate analysis confirmed the value of osteopontin expression as an independent prognostic factor (*p* < 0.0001) [49].

## 3. Mesothelin

Mesothelin is one of the most extensively studied MM biomarkers and is the only blood-based biomarker approved by Food and Drug Administration in MM diagnosis [51]. Mesothelin, also known as MSLN, is a protein that in humans is encoded by the *MSLN* gene that is expressed in mesothelial cells [51]. In the literature we often refer to mesothelin as SMRP (Soluble Mesothelin Related Peptides), or soluble mesothelin related proteins; these proteins are found in normal mesothelium cells and are overexpressed in various tumors [51]. These are membrane-bound peptides that can be processed to produce megakaryocyte enhancement factor (MPF) and mesothelin, which remains attached to the cell membrane by binding to glycophaphtidininositol [51,52,53]. Further studies have shown that mesothelin promotes tumor cell survival and proliferation through NF-κB pathway activation, resulting in an increase of interleukin-six level [52,54]. Hollevoet and colleagues have shown that mesothelin has a high specificity, equal to 96%, but only a low sensitivity of only 47% [55]. As to the prognosis, the results are still conflicting. Several studies have shown no correlation between serum mesothelin level and free survival illness or global [54].

Tian, L. and colleagues carried out a meta-analysis to determine prognostic significance of soluble mesothelin in MM [54]. In this document, hazard ratio with 95% CI was used to evaluate the prognostic value of soluble mesothelin and the effect of clinicopathological features on the survival of MM. Eight eligible studies involving 579 patients were selected for this meta-analysis. The results showed that soluble mesothelin level was significantly correlated with the survival of MM (pooled HR (Hazard Ratio): 1.958, 95% CI: 1.531–2.504, *p* = 0.000). In addition, the survival of MM was significantly correlated with some clinicopathological characteristics such as tumor histology (HR = 3.214, 95% CI = 2.071–4.988, *p* = 0.000) and tumor stage (HR = 2.007; 95% CI = 1.477–2.727).

Others, however, have shown that at threshold values of 1 and 3.5 nmol/L, SMRP levels are inversely related to the overall survival of patients with MM [54,55,56,57,58,59,60].

The possible explanations for the conflicting results regarding the use of mesothelin as a prognostic marker include small sample size and heterogeneous treatment between the different studies. Therefore, studies with more standardized treatments and involving a greater number of patients are necessary to better understand the role of SMRP as a prognostic biomarker.

## 4. Fibulin-3

Fibulin-3 (Fb-3, also known as EFEMP1 (EGF containing fibulin extracellular matrix protein 1)) is an extracellular glycoprotein generally expressed in most tissues already in the early embryonic stage. It is one of the seven proteins belonging to fibulins family [61,62]. Fibulins are characterized by an arrangement in pairs of epidermal growth factor-like (EGF-like) domains calcium binding and a fibulin-type C-terminal module. Fb-3 is encoded by the *EFEMP1* gene (also known as S1-5) located on chromosome 2p16 [61,62,63].

In adults, Fb-3 is widely distributed in different tissues, including the eye. In particular, there is a high expression of this glycoprotein in epithelial and endothelial cells on the basal membrane level [61,62]. They are very important structures not only for structural or filtering functions, as for example in renal glomerulus, but also because they come into play to determine cell polarity and regulation of metabolism, proliferation, differentiation and migration cells [61,62].

The first study proposing the use of Fb-3 as a possible MM biomarker was conducted by Pass et al. in 2012 [64]. The intent of the study was to analyze the reliability of Fb-3 compared to mesothelin, a protein widely studied as a biomarker. In this study, the authors, after studying a sample consisting of 92 MM and 290 controls (ex-exposed to asbestos, subjects with benign and malignant pleural effusions not from MM, other tumors and not exposed healthy subjects) showed a high diagnostic accuracy of this marker (AUC = 0.99) with sensitivity (0.97) and specificity (0.95) greater than those commonly found for mesothelin. The analysis restricted to stage I and II MM (28 cases) and to controls exposed to asbestos confirmed a high diagnostic accuracy (AUC = 0.99; sensitivity = 1.00; specificity = 0.94). The validation of this data in a sample external to the one under study, however, led to a reduction in AUC (0.87) and a significant reduction in sensitivity (0.73).

In spite of the encouraging results obtained by the study of Pass et al., subsequent experiments have provided conflicting results. A cohort of 153 patients (82 of whom with MM) was studied by Creaney et al. [65], who reported a sensitivity of 22% and a specificity of 95% for plasma Fb-3 (cut-off: 52 ng/mL AUC = 0.671). These values were definitely lower than those obtained, on the same patients, for mesothelin (sensitivity 56%, specificity 95%—AUC = 0.816), which, on the contrary, seems to provide a much higher diagnostic precision on the plasma samples. Although in this study mesothelin was superior to Fb-3 from a diagnostic viewpoint, the authors considered the latter superior from the prognostic point of view. Indeed, high Fb-3 values correlated negatively with the patients’ prognosis [65]. A possible explanation of this data could derive from the increased expression of Fb-3 by the biphasic and sarcomatoid histotypes which are generally characterized by a worse prognosis [66].

Overall, studies suggest that Fb-3 could play a role in the development of neoplastic and non-neoplastic diseases of the respiratory tract in subjects exposed to asbestos and/or asbestos-like fibers [67,68,69,70,71,72,73]. Furthermore, some studies are investigating the hypothesis that Fb-3 may be responsible for the malignant transformation of mesothelial cells after exposure to fibers.

## 5. HMGB1 (High Mobility Group Box 1)

Recently, interesting results have been reported for the High Mobility Group Box 1 (HMGB1) protein [74]. This is a protein belonging to the high mobility group of proteins that is normally abundant in the nucleus [74]. Once acetylated, it can accumulate in the cytoplasm and subsequently be released by monocytes and macrophages in the extracellular matrix where it plays an important pro-inflammatory role [74,75]. Recent studies have shown that exposure to asbestos leads to necrosis of mesothelial cells, resulting in the release of HMGB-1, which binds to its main receptor and causes the activation of Nalp3 inflammosome and IL-1b secretion [74,75,76,77]. This inflammatory cascade has been linked to the carcinogenesis promoted by asbestos. Scientific studies have shown that HMGB1 serum levels are higher in MM patients than in asbestos-only controls (not affected by MM) [78]. Furthermore, if a threshold value of 9 ng/mL is given, there is a significant negative correlation between the HMGB1 serum level and survival, which would seem to indicate HMGB1 as a potential prognostic biomarker [79]. Napolitano et al. also demonstrated that the total level of HMGB1 in the blood was significantly higher in patients with MM and in patients exposed to asbestos compared to healthy controls [75]. Specifically, at a threshold of 2.0 ng/mL the authors showed that HMGB1 hyperacetylated in the serum, had sensitivity and specificity of 100% in differentiating patients with MM compared to subjects exposed to asbestos and healthy controls [75].

These results therefore suggest a role for hyperacetylated HMGB1 as a potential diagnostic [75] and prognostic [79] marker in the recognition of those potentially affected by MM.

## 6. Aquaporin-1

Aquaporins (AQPs), are a family of at least 13 transmembrane channel proteins that facilitate the flow of water molecules and represent a potential target for cancer therapy [80,81]. Aquaporin-1 (AQP1) was initially identified on cell membranes of erythrocytes in 1988 and its classic role in facilitating trans-cellular flow of water has been widely studied and well understood [81]. The subsequent analysis showed that AQP1 is more than a simple water channel, its involvement in cell migration, in fat metabolism, in the migration of leukocytes and in the neural signal transduction has revealed an important role in the cancer physiopathology, obesity, immune system cells dysfunction and epilepsy [81,82]. AQPs, therefore, play a role in normal cellular water transport processes, but also in cell proliferation and pain perception, whereas in cancer, the expression of aquaporins has been shown to play a role in the growth and metastatic potential of different tumors, including pulmonary adenocarcinoma [83]. MM grows characteristically by direct diffusion along the surface of the pleura, where it forms nodules on the pleural surface; it is thought that this growth mode refers to the sliding movement of the tumor cells. Aquaporins facilitate the movement of both endothelial cells and some tumor cells.

The AQP1 was found in pleural and peritoneum MM, and a role of AQP1 was found in the transport of water from the pleura through the mesothelial cells in a mouse knockout model [84,85,86]. AQP1 can be expressed by vascular endothelium just like by cancer cells, and blocking aquaporin expression in both tumor cell types may be useful for prognostic purposes [85,86]. It has been suggested that the modulation of tumor cells, either by specific aquaporin inhibitors or by agonists or by indirect modulation of closely associated growth factors, may turn out to be a future treatment strategy for some cancers. The expression of AQP1 in MM tumor cells is an independent prognostic factor to improve survival time in MM, high levels of AQP1 expression of MM tumor cells predict an increase in survival [87,88] while in other tumors the increase of AQP1 levels is associated with worse prognosis, including breast cancer, melanoma, urothelial carcinoma and pharynx squamous cell carcinoma [81]. It has been hypothesized that the highest expression of aquaporin in MM may reflect better differentiation, since the normal mesothelium expresses aquaporin.

## 7. Perspective: Protein & Molecular Biomarkers?

MM is still today a highly aggressive tumor characterized by a late diagnosis that determines a worsening of the prognosis.

The appearance of this neoplasia is typically related to occupational exposure to asbestos fibers and/or environmental exposure to asbestiform fibers present in any geographical areas, such as the Fluoro-edenite fibers present in Biancavilla (Sicily) [89,90], the eronite mineral fibers found in Anatolia (Turkey) [10] and the tremolite mineral fibers present in New Caledonia [24,25,26].

Hence, the need arose to identify non-invasive protein indicators, usable in the field of secondary prevention, for an early diagnosis of MM, in order to diagnose the disease in a phase in which the surgery and radio chemotherapy may be more effective in increasing survival. Although the literature is rich in studies that have evaluated old and new biomarkers (an overview of the protein biomarkers for MM is presented in Table 1), none of the current available markers is suitable for an early diagnosis of MM in subjects exposed to asbestos. Taking into account the heterogeneity of MM, it could be strategically important to introduce new markers such as microRNA (miRNA) (see Table 2).

miRNAs are a family of small non-coding RNAs, about 21–25 nt long, that contribute to regulating gene expression [91]. In recent years, the use of miRNAs has been proposed as biomarkers for various neoplastic pathologies, including pleural MM [91,92]. Several research groups analysed and compared circulating miRNAs in serum samples from patients with MM, workers exposed to asbestos and healthy subjects [93,94,95,96,97,98,99,100].

Currently, literature proposes the stratification of high-risk subjects and an early diagnosis of MM through the perusal of the following pool of blood analysis: miRNA-126-3p, miRNA-625-3p and miRNA-103a-3p in pairing with mesothelin and fibulin-3. Instead, for MM patients, the analysis of miRNA-16-5p, miRNA-126-3p, miRNA-143-3p, miRNA-145-5p, miRNA-192-5p, miRNA-193a-3p, miRNA-200b-3p, miRNA-203a-3p and miRNA-652-3p might be useful to monitor sensitivity to therapy and for prognostic purposes [92,94,95,96,97]. The scientific community, however, constantly updates the miRNA which can be associated with MM early diagnosis and prognosis.

Mozzoni and collegues recently carried out a study to identify a pattern of miRNAs as possible diagnostic biomarkers for patients with MM and asbestosis and highlighted that miRNA-16, miRNA-17, miRNA-126, and miRNA-486 were significantly lower in patients with MM and asbestosis than in controls; in particular, miRNA-16 was directly related to MM patient prognosis, suggesting its possible use as a prognostic marker in MM patients [101].

Other studies revealed that several miRNAs are involved in deregulation and in all molecular mechanisms associated with MM development [102,103].

It could be particularly helpful to use a combination of several protein markers and miRNAs to improve diagnostic accuracy, instead of using single markers. Despite intense research, the translation of these results from research to clinical practice remains problematic.

## Figures and Tables

**Table 1:** Protein biomarkers for early diagnosis and prognosis of Malignant pleural mesothelioma (MM).

Protein	Description	Analysis
Osteopontin	Encoded by the *SPP1* gene (secreted phosphoprotein 1), which mediates cell-matrix interaction and cell signaling through interaction with integrin and CD44 receptors [44,45].	Serum-Tissue
Mesothelin	Encoded by the *MSLN* gene, that is expressed in mesothelial cells [51].	Serum-Tissue
Fibulin-3	Encoded by the *EFEMP1* gene (also known as S1-5) located on chromosome 2p16 [61,62,63].	Serum-Bronchial Aspirates-Tissue
HMGB1	Encoded by the *HMGB1* gene [74].	Serum-Tissue
Aquaporin-1	Encoded by the *AQP1* gene [81].	Serum-Tissue

**Table 2:** Potential miRNA biomarkers for Malignant pleural mesothelioma (MM).

mRNAs in MM	Clinical Utility	Sample Analysis
miRNA-126-3p	early diagnosis	Serum
miRNA-625-3p		Serum
miRNA-103a-3p		Serum
miRNA-16-5p	Prognosis	Serum-Tissue
miRNA-126-3p		Serum-Tissue
miRNA-143-3p		Serum-Tissue
miRNA-145-5p		Serum-Tissue
miRNA-192-5p		Serum-Tissue
miRNA-193a-3p		Serum-Tissue
miRNA-200b-3p		Serum-Tissue
miRNA-203a-3p		Serum-Tissue
miRNA-652-3p		Serum-Tissue
